# Weight Loss or Liver Loss: A Case Report on Fulminant Hepatic Failure Secondary to Garcinia cambogia Supplementation

**DOI:** 10.7759/cureus.41778

**Published:** 2023-07-12

**Authors:** Danny Le, Brody A Hydro, Chase L Jones, Gillian A Beauchamp

**Affiliations:** 1 Department of Emergency and Hospital Medicine, Lehigh Valley Health Network/University of South Florida (USF) Morsani College of Medicine, Allentown, USA; 2 Department of Emergency and Hospital Medicine, Division of Medical Toxicology, Lehigh Valley Health Network/University of South Florida (USF) Morsani College of Medicine, Allentown, USA

**Keywords:** toxicity management, n-acetylcysteine, hepatitis, herbal supplementation, liver failure

## Abstract

This case describes a 56-year-old man with a past medical history including sickle cell trait requiring blood transfusions, who presented to the emergency department (ED) with generalized weakness and fatigue following *Garcinia cambogia* supplementation. Initial laboratory abnormalities included: aspartate aminotransferase (AST) and alanine transaminase (ALT) 4,222 U/L and 4,664 U/L respectively, alkaline phosphatase 215 U/L, international normalized ratio (INR) 3.2, and his model for end-stage liver disease was 37. Creatinine, hemoglobin and hematocrit, and ferritin levels were all elevated. The differential diagnosis for his acute illness was broad ranging from hemochromatosis, anabolic steroid use, and portal venous thrombosis. The patient was started on N-acetylcysteine (NAC) and his liver function improved. He was discharged on hospital day 10 and instructed to discontinue his supplements and follow up for repeat blood work. This case explores the critical management of *G. cambogia* toxicity. The patient explored *G. cambogia *as an herbal supplementation resulting in weight loss, worsening generalized fatigue, and fulminant hepatic failure.

## Introduction

The United States prevalence of obesity in adults from 2017 to 2020 was 42% [1]. Weight-loss methods include exercise, fad diets, and herbal supplementation. As a result, a multi-billion-dollar industry targets weight-loss enthusiasts and health-conscious individuals [2]. *Garcinia cambogia*, an herbal product derived from the Malabar tamarind tree, is often touted as a weight-loss pill despite limited FDA oversight [3]. However, there is emerging evidence that links *Garcinia cambogia* use to liver failure [4]. We report a case of *Garcinia cambogia* supplementation leading to fulminant hepatic failure.

## Case presentation

A 56-year-old man with a past medical history of sickle cell trait requiring blood transfusions presented to the emergency department (ED) with generalized weakness and fatigue for two weeks. About seven months prior, he ran out of Anadrol-50, which he was using to supplement his health. Since then, he had been feeling more fatigued which prompted him to explore natural approaches to addressing his symptoms including fasting, and herbal supplements including *Garcinia cambogia*, alpha lipoic acid, and Boost™ Protein shake. Upon initiation of supplementation with *G. cambogia*, he noted weight loss of about 10 pounds in a span of two weeks and gradually worsening generalized fatigue, which prompted his visit to the ED.

Initial vital signs were blood pressure (BP) 134/88 mmHg, heart rate (HR) 84 BPM, temperature 98.5 degrees F, respiratory rate (RR) 18, and oxygen saturation (SpO2) 98%. His physical exam was remarkable for a thin man with scleral icterus, mild abdominal pain, especially in the right upper quadrant, as well difficulty with word finding and confusion. His laboratory studies are shown in Table 1 and include the following notable abnormal results: international normalized ratio (INR) 3.2, while not on any anticoagulants, serum creatinine (Cr) 2.73mg/dL, hyponatremia 127 mmol/L, hypochloremia 91 mmol/L, hemoglobin (Hgb) 20.5 g/dL, polycythemia, model for end-stage liver disease (MELD) score 37, and his white blood cells were within normal limits.

**Table 1:** Patient’s laboratory results pCO2: partial pressure of carbon dioxide

Laboratory tests	Results	Reference ranges
Aspartate aminotransferase (AST)	4,222 U/L	7-52 U/L
Alanine transaminase (ALT)	4,664 U/L	13-39 U/L
Alkaline phosphatase	215 U/L	34-104 U/L
International normalized ratio (INR)	3.2	< 1.5
Lactate	11.6 mmol/L	0.5-2.2 mmol/L
Bilirubin	5.8 mg/dL	0.2-1.1 mg/dL
Creatinine	2.73 mg/dL	0.70-1.30 mg/dL
Sodium	127 mmol/L	136-145 mmol/L
Chloride	91 mmol/L	100-108 mmol/L
Anion gap	23	4-18
Bicarbonate (HCO3-)	12 mmol/L	21-37 mmol/L
Venous blood gas (VBG)	7.14	7.31-7.41
pCO2	46 mmHg	41-51 mmHg
Hemoglobin	20.5 g/dL	12.5-17.0 g/dL
Hematocrit	62.5%	37.0- 48.0%
Red blood cell (RBC)	7.14 mill/cmm	4.00-5.40 mill/cmm
Platelet count	77 thou/cmm	140-350 thou/cmm
Ferritin	>12000 ng/mL	22-322 ng/mL

Given those laboratory results, the ED consulted the medical toxicology team who recommended starting intravenous N-acetylcysteine (NAC) (until liver enzymes were less than 1000 U/L) and vitamin K for his coagulopathy (though his acetaminophen level was negative). He was transferred from an outside hospital to the intensive care unit due to moderate metabolic acidosis with venous blood gas (VBG) of 7.14 and lactate of 11.6 in addition to his hepatic failure. Hepatology, hematology, and nephrology were consulted for his multi-organ pathology. The differential diagnosis for his acute illness was broad ranging from hemochromatosis, anabolic steroid use, and portal venous thrombosis. After the initiation of NAC, the patient’s liver function started to improve. He was continued on an infusion of NAC for 5 days until his aspartate aminotransferase (AST) and alanine transaminase (ALT) were less than 1,000 U/L. He was managed with a bicarbonate infusion for his high anion gap metabolic acidosis at 75mL/hr over the course of 4 days until bicarbonate normalized. He was evaluated with multiple imaging modalities including a portal/hepatic ultrasound, magnetic resonance imaging (MRI), magnetic resonance cholangiopancreatography (MRCP), and nuclear medicine gallbladder which demonstrated chronic cholecystitis but no acute process. Genetic markers for hemochromatosis were also performed which were negative. Similarly, a hepatitis panel including cytomegalovirus, Epstein-Barr virus, hepatitis A, B, and C were also negative. While his stay was complicated by the development of atrial flutter due to his acute illness, his laboratory derangements improved with the appropriate interventions, and he was discharged 10 days later with instructions to discontinue his supplements and follow up for repeat blood work. One month later, he went to his primary care provider’s office and had a repeat comprehensive metabolic panel (CMP) which showed the resolution of his initial lab changes. His AST and ALT were 16 and 19, his alkaline phosphatase was 110, and his creatinine was 1.22. His hemoglobin and hematocrit also improved, and he no longer had polycythemia.

## Discussion

*G. cambogia* has been used for centuries as an appetite suppressant [5]. The main contributing component is (-)-hydroxycytric acid (HCA). HCA acts as a competitive inhibitor of the adenosine triphosphate (ATP)-citrate lyase enzyme which converts citrate into acetyl-coenzyme A (CoA), decreasing fatty acid biosynthesis and lipogenesis, thus decreasing weight gain. In addition, it also stimulates liver gluconeogenesis along with glycogen storage and upregulates serotonin, stimulating satiety [4]. These biochemical processes have been beneficial for weight loss, especially with significant visceral and subcutaneous adipose reduction [3].

Despite its popularity and success, there have been multiple case reports illustrating the potentially catastrophic effects of *G. cambogia* on the human body as seen in this case report [6]. In certain animal studies, the introduction of HCA destroys the integrity of the liver through steatohepatitis leading to significant necrosis [7]. One of the proposed mechanisms is the upregulation of certain inflammatory cytokines including tumor necrosis factor alpha (TNF) [8]. While supplementation with *G. cambogia* can lead to fat-burning and weight loss via upregulation of TNF leading to the catabolism of certain adipose tissues, it also leads to a proinflammatory state which contributes to hepatitis and eventual fulminant hepatic failure [8]. This is further exemplified in *G. cambogia*-poisoned individuals who required liver transplant after developing fulminant liver failure where over 70% of the diseased liver hepatic parenchymal cells depicted submissive necrosis [9].

## Conclusions

There is currently no gold standard treatment for *Garcinia cambogia* induced liver toxicity except for cessation. Therefore, it is imperative not only for consumers but also medical professionals to be aware of the different types of herbal supplements available and their adverse effects. A detailed history may lead to the correct diagnosis, rapid intervention, and overall better patient outcomes. As in this case, cessation of use of *G. cambogia* and supportive care are critical steps in the management of toxicity.
